# Evolution of Pathogenesis and Trends in the Treatment of Melasma in Last Two Decades

**DOI:** 10.1111/jocd.16758

**Published:** 2025-01-02

**Authors:** Rashmi Sarkar, Aanchal Bansal, Michael Gold

**Affiliations:** ^1^ Department of Dermatology, Venereology and Leprosy Lady Hardinge Medical College and Hospital New Delhi India; ^2^ Gold Skin Care Center Tennessee Clinical Research Center Nashville Tennessee USA

**Keywords:** melasma, trends, updates in treatment

Melasma is a common relapsing pigmentary disorder that presents with symmetrically patterned hyperpigmented patches in the photo‐exposed regions of the face such as the centro‐facial, malar, and mandibular region. The first introduction of the disease was described in 1910. For decades, there has been a change in understanding of the etiopathogenesis and evolution of treatment in melasma. Syder and Elbuluk [1] reviewed and summarized the history of melasma from its origin till the early 2000s and described that melasma is a multifactorial disease with a phenotypic presentation related to the interplay of genetic predisposition, oxidative stress, hormonal factors and light exposure. Further development and research in the past decade have elucidated other pathogenetic mechanisms.

The genetic origin has been described with an increased predisposition of the disease by 41%–61% in first‐degree relatives, as compared to 16%–28% in the normal population, in a Brazilian study [2]. It has been recently found to follow an autosomal dominant inheritance pattern. The presence of senescent fibroblasts in melasma skin leads to increased release of inflammatory and melanogenic factors. Esposito et al. [3] found lower cell density, altered morphology, higher amount of cytoplasmic senescence‐β‐galactosidase with altered expression of genes in fibroblasts of melasma skin as compared to normal photo‐exposed regions. The most important factors attributed to melasma development and aggravation are unsatisfactory use of sunscreens, incomplete blockage from solar radiations involved in melanogenesis including ultraviolet A and B rays, visible light and association with cooking heat and occupational exposure [4]. Recent literature talks about molecular signs of oxidative stress such as increased expression of p38 protein kinase in the upper dermis in response to various stimuli, such as stress, inflammatory cytokines, ultraviolet rays, heat, and osmotic shock [5].

The development of pathological understanding has broadened the therapeutic options over recent years and treatment can be tailored according to individualistic requirements.

A review of recent trends in the treatment of melasma by Zheng et al. [6] in this journal highlighted the centrality of topical treatments like hydroquinone in combination with tretinoin and topical corticosteroids as triple combination creams in the early 2000s. Other topical agents like azelaic acid, glycolic acid and retinoic acid were also frequently used for their exfoliating and depigmenting properties. The early decade highlighted an enormous trend of chemical peel treatment with glycolic acid peels (20%–70%) and trichloroacetic acid peels to treat epidermal melasma (Figure 1). Peels were often recommended as a second‐line therapy, especially for patients who did not respond to topical treatments [7]. However, in the mid‐2000s to early 2010s, there was an advancement in the development of laser treatments such as Q‐switched neodymium‐doped yttrium aluminum garnet laser (QSNY), Intense pulsed light (IPL) for the treatment of patients particularly not responding to topical and chemical treatments. However, the risk of development of post‐inflammatory hyperpigmentation (PIH) limited its use in patients of skin of color. In the mid‐2010s (2012–2018), tranexamic acid (TXA) gained popularity because of its efficacy and safety profile across various skin types in the treatment of resistant melasma. TXA can be administered orally, topically, or via intradermal injections. TXA is an antifibrinolytic agent, that blocks the conversion of plasminogen to plasmin and exhibits anti‐inflammatory action by inhibiting of release of arachidonic acid leading to decreased synthesis of prostaglandins and fibroblast growth factors thereby, inhibiting melanin synthesis. The description of the first use of TXA in melasma was reported by Nijor in 1979 [8]. Since its development, it has been researched extensively and has been found effective orally at a dose of 250 mg twice daily. The largest retrospective analysis of its use in the South Asian population published by Lee, Thng, and Goh [9] reported it as an effective monotherapy with visible effects within 2 months, a 27% relapse rate and adverse events reported in 7.1% of cases with gastrointestinal symptoms predominantly.

**FIGURE 1:**
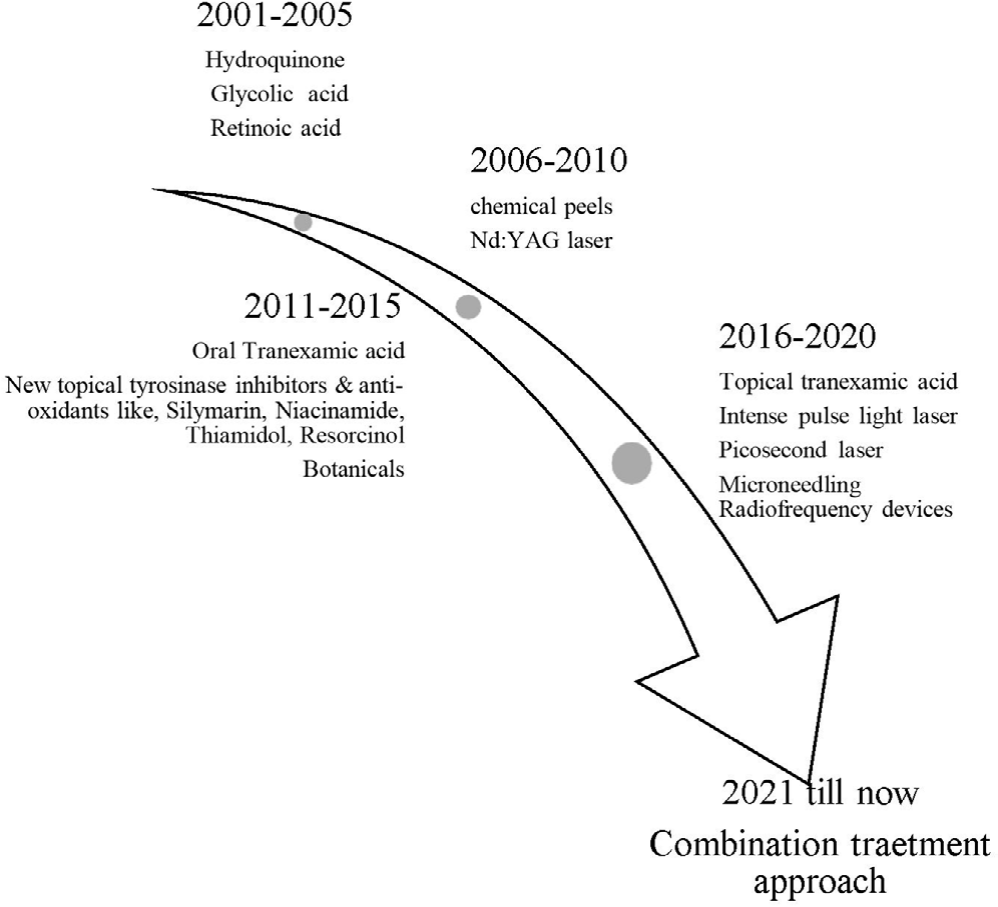
Evolution of treatment trends in last two decades.

In the subsequent years (2015–2020), the trend shifted toward a multimodal approach of combining various treatment options. Laser therapy continued to evolve, with newer devices like the picosecond laser gaining attention for their lower risk of side effects compared with traditional lasers. The picosecond laser showed effectiveness in treating melasma without causing significant PIH. The last decade's narrative also focuses on developing safe and efficacious tyrosinase inhibitors for the treatment of epidermal melasma. Cysteamine has been explored widely for its role in melasma because of its additional antioxidant properties by inhibition of peroxidase enzyme. Dos Santos‐Neto et al. [10] published a meta‐analytic study of the effectivity of 5% cysteamine in melasma with mild and reversible side effects. Another agent, thiamidol 0.2% (isobutylamido thiazolyl resorcinol) with potent tyrosinase inhibition activity, has shown equivalent efficacy as compared to 4% hydroquinone in reduction of melasma score when applied in a double‐layered technique [11]. Newer treatment modalities now focus on the increased transepidermal delivery of drug molecules for achieving adequate therapeutic response of drug. The ethosomal, niosomal, and liposomal formulations entrapping the depigmenting agents have better solubility, thereby increasing the transcellular permeation of the drug leading to improved skin melanin content, skin elasticity and moisture content [12, 13].

In the recent years (2020–2023), there is a shift in the treatment paradigm of chemical peels as they are now preferred in combination with other procedures like microneedling to enhance the results. Microneedling is a semi‐invasive office procedure that works by creating micro‐perforations through microneedles and delivery of topical drugs transdermally. It is often combined with platelet‐rich plasma (PRP) therapy for its regenerative properties, promoting collagen production and aiding in the repair of damaged skin. The addition of depigmenting agents such as vitamin C, glutathione, tranexamic acid, N‐acetyl glucosamine led to better improvement [14]. Mekawy, Sadek, and Seddeik Abdel‐Hameed [15] revealed similar efficacy of fractional CO_2_ and microneedling in the delivery of tranexamic acid and improvement of melasma. The recent trend in laser treatments has shifted toward low‐fluence Q‐switched lasers and fractional lasers, which offer more targeted treatment with fewer risks of side effects. A review study published by Lee et al. [16] on the role of Q‐switched Nd:Yag (QSNY) laser in melasma analyzed 42 studies, elucidating the mechanism of low‐fluence QSNY as sub‐cellular photo‐thermolysis leading to a decrease in pigmentation and showing favorable outcomes in majority studies, particularly in Asian individuals.

Recent literature has been found to focus more on the combination approach in a step‐wise or sequential manner and counseling of patients regarding treatment adherence for achieving prolonged remission. Tranexamic acid continues to be a major focus of melasma research, particularly in oral and topical formulations. Its success in treating resistant melasma, combined with a lower side effect profile, has cemented its place as a key treatment option. The consensus focuses on the formulation of an effective treatment plan consisting of topical agent and systemic drug in cases of progressive disease with adequate sun protection for induction, followed by the addition of procedural treatment like lasers, microneedling, PRP therapy for unresponsive cases and maintenance of remission after adequate response with suitable topical agent in long‐term to prevent relapses.

## Conflicts of Interest

The authors declare no conflicts of interest.

## Data Availability

The authors have nothing to report.
